# Supplementary material for: A mechanically adaptive hydrogel with a reconfigurable network consisting entirely of inorganic nanosheets and water
Source: Nat Commun. 2020 Nov 27;11:6026. doi: 10.1038/s41467-020-19905-4 (PMC7699623; doi:10.1038/s41467-020-19905-4)
Supplement: Supplementary file 1 — Supplementary Information [file 41467_2020_19905_MOESM1_ESM.pdf]

Supplementary Information

A mechanically adaptive hydrogel with a reconfigurable network consisting entirely of inorganic nanosheets and water

Koki Sano*, Naoki Igarashi, Yasuo Ebina, Takayoshi Sasaki,
Takaaki Hikima, Takuzo Aida*, and Yasuhiro Ishida*

*To whom correspondence should be addressed.
E-mail: koki.sano@riken.jp (K.S.); aida@macro.t.u-tokyo.ac.jp (T.A.); y-ishida@riken.jp (Y.I.)

Table of Contents

Supplementary Methods S2

Supplementary Figures (Supplementary Figs. 1–6) S3

Supplementary References S9

Supplementary Methods

General: Centrifugation was conducted by using a TOMY model CAX-571 centrifuge with a TOMY model CA-16 rotor. Rheological and compression measurements were performed by using an Anton Paar model MCR-301 rheometer. Polarized optical microscopy was performed on a Nikon model Eclipse LV100POL optical polarizing microscope or a KEYENCE model VHX-5000 digital microscope, with controlling the temperature by using a Linkam model 10030 temperature controller. Free-ion concentrations were estimated from ion conductivities by using a Horiba model DS-71E conductivity meter. Zeta potential and dynamic light scattering (DLS) measurements were performed by using a Malvern model Zetasizer Nano ZSP. Differential scanning calorimetry (DSC) was recorded on a Mettler model DSC30 calorimeter. Scanning electron microscopy (SEM) was performed on a HITACHI model SU8010. Transmission electron microscopy (TEM) was performed on a JEOL model JEM-1230 operated at an accelerating voltage of 80 kV. UV/Vis absorption spectroscopy was conducted on a JASCO model V-670 UV/VIS/NIR spectrophotometer using a 1mm-thick quartz cuvette.

Materials: An aqueous dispersion of unilamellar titanate(IV) nanosheets (TiNSs; Fig. 1a and Supplementary Fig. 1) was prepared according to the literature method[1]. Dispersions containing chopped TiNSs (Fig. 6d) were prepared by sonicating the as-prepared TiNS dispersion with a QSONICA model XL-2000-600 homogenizer, where the sizes of TiNSs were controlled by tuning the sonication intensity and time. The hydrodynamic sizes of TiNSs were estimated by DLS measurement. Water was obtained from a Millipore model Milli-Q integral water purification system. All reagents were used as received from Sigma-Aldrich [thiol-appended polyethylene glycol (average molecular weight: 6 kDa) and hydrogen tetrachloroaurate(III) hydrate ($HAuCl_4 \cdot 3H_2O$)] and Wako [40% tetrabutylammonium hydroxide ($(C_4H_9)_4N^+OH^-$) aqueous solution and sodium citrate].

Supplementary Figures (Supplementary Figs. 1–6)

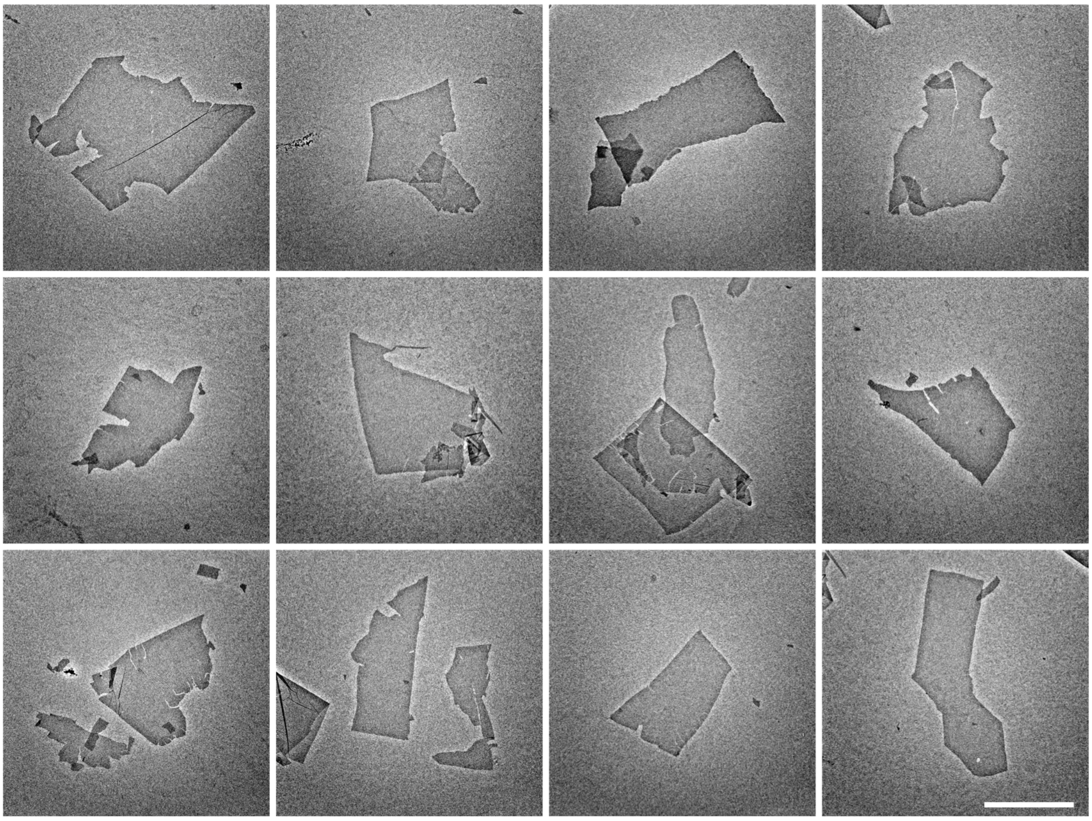

Supplementary Fig. 1 | Transmission electron microscopy (TEM) images of TiNSs.

TiNSs were deposited on a hydrophilized carbon-covered copper grid. Scale bar: 5 μm.

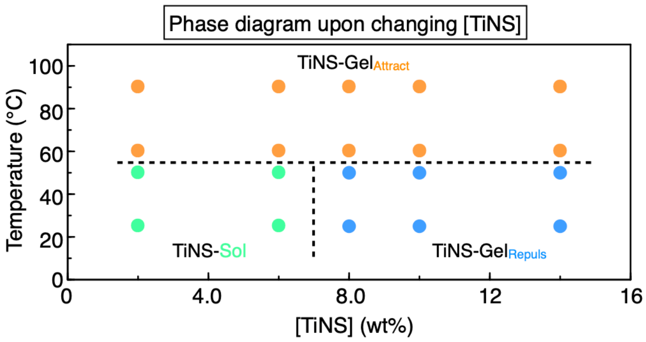

Supplementary Fig. 2 | Phase diagram of TiNS-Gel upon changing [TiNS].

Thermal phase diagram of TiNSs dispersed in water with systematically changing [TiNS].

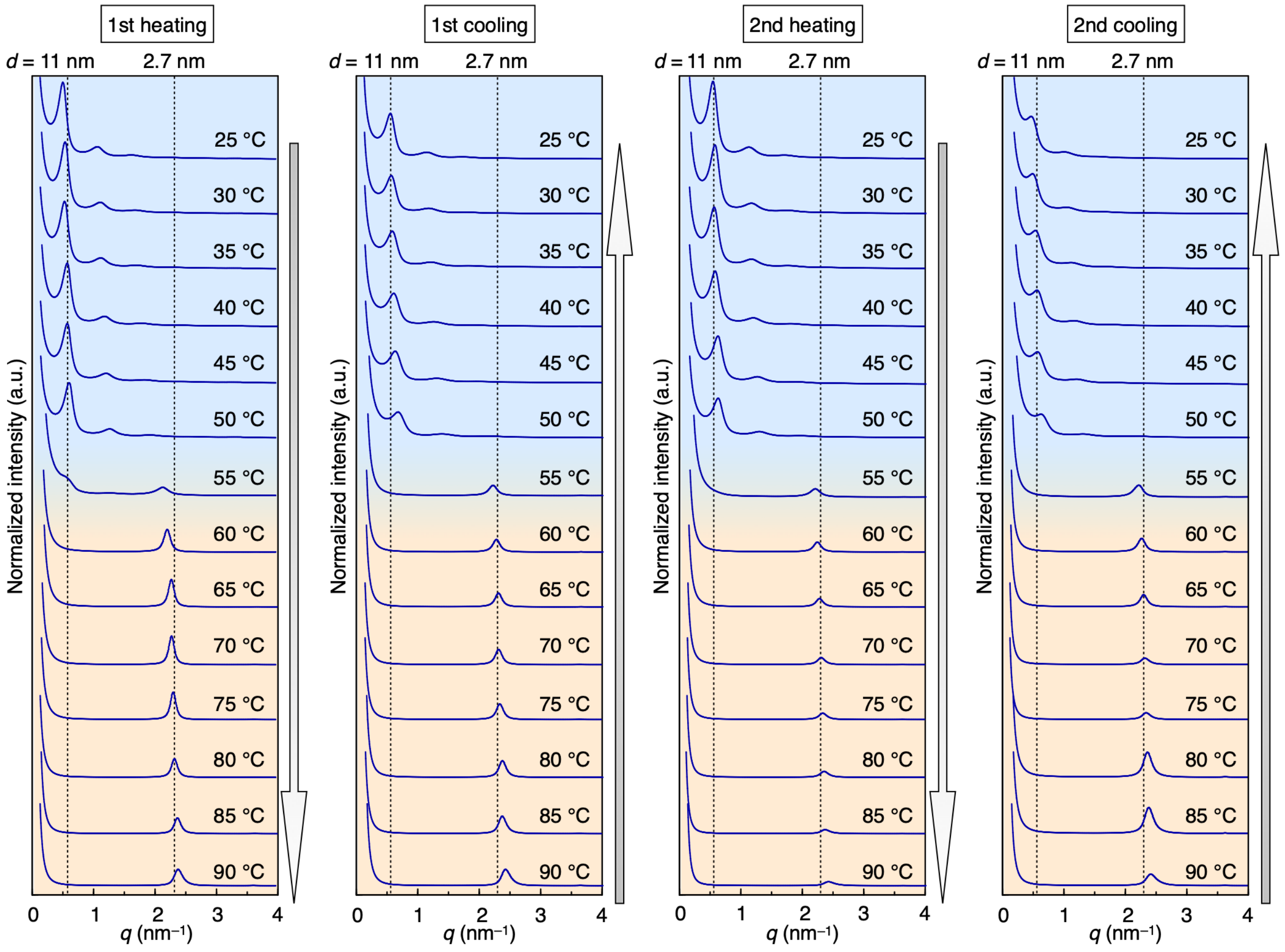

Supplementary Fig. 3 | SAXS analysis of TiNS-Gel at different temperatures.

1D SAXS profiles of TiNS-Gel ([TiNS] = 14 wt%) upon repeated thermal scanning between 25 and 90 °C.

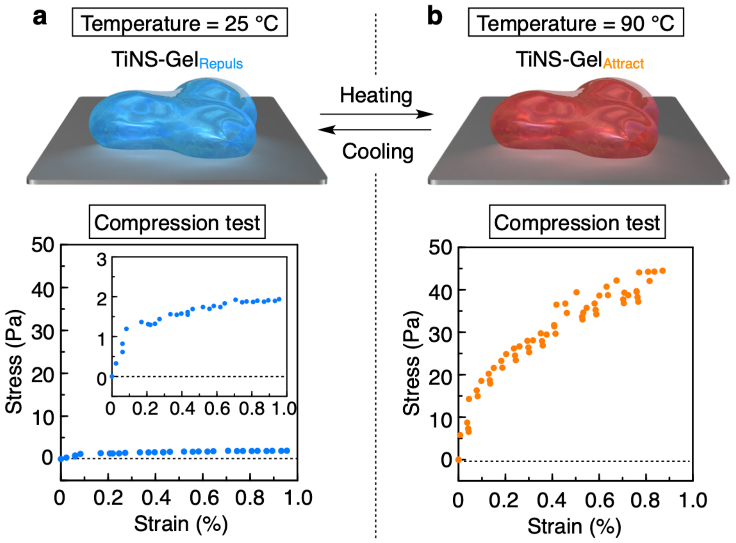

Supplementary Fig. 4 | Compression tests of TiNS-Gel through the transition between TiNS-Gel$_{\mathbf{Repuls}}$ and TiNS-Gel$_{\mathbf{Attract}}$.

a, **b**, Strain–stress curves of TiNS-Gel ([TiNS] = 14 wt%) upon compression with keeping temperature at 25 (**a**) and 90 °C (**b**).

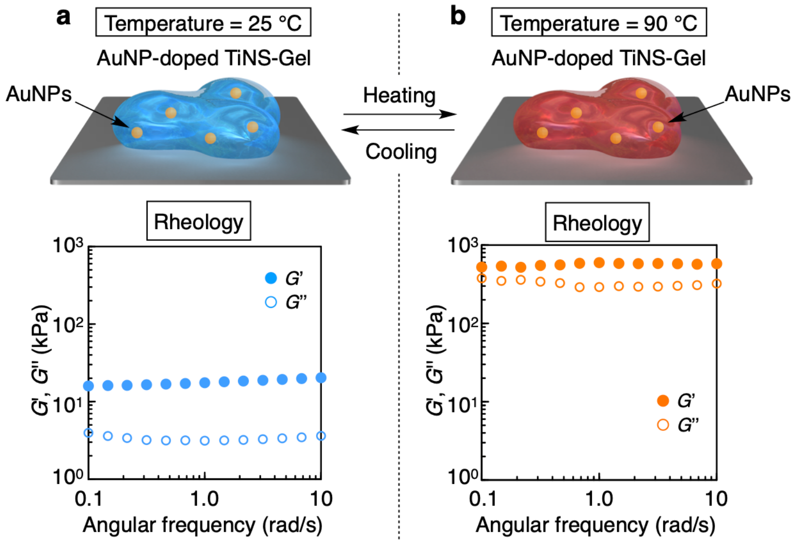

Supplementary Fig. 5 | Rheological tests of AuNP-doped TiNS-Gel through the transition between TiNS-Gel$_{\mathbf{Repuls}}$ and TiNS-Gel$_{\mathbf{Attract}}$.

a, **b**, Storage (G') and loss (G'') moduli on frequency sweep (0.1–10 rad s^{-1}) at a fixed strain (0.1%) of AuNP-doped TiNS-Gel ([TiNS] = 14 wt%; [AuNP] = 0.05 wt%) with keeping the temperature at 25 (**a**) and 90 °C (**b**).

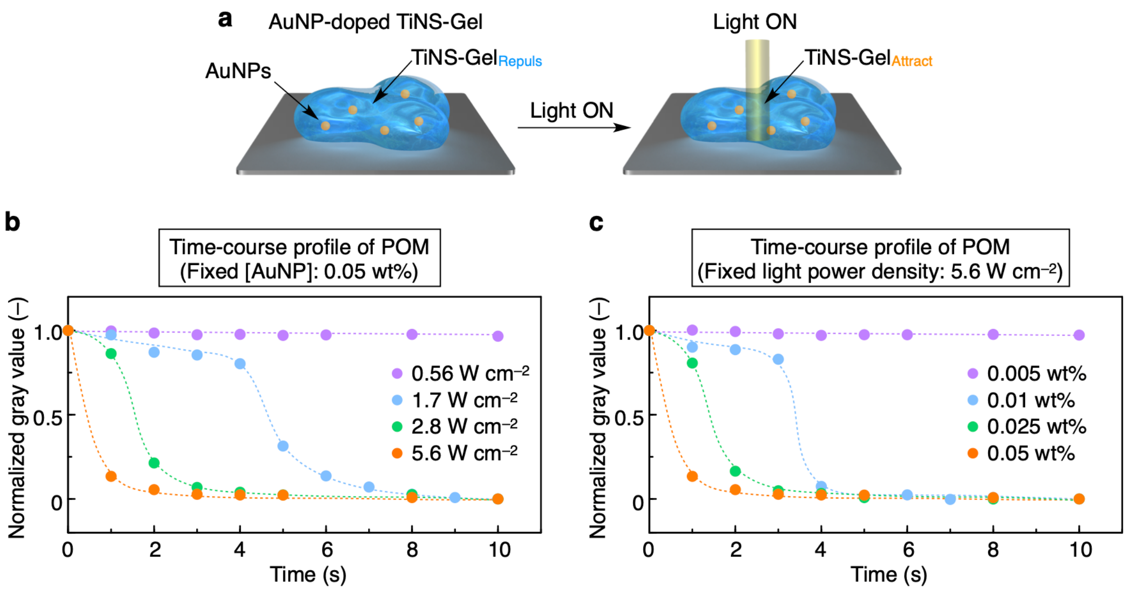

Supplementary Fig. 6 | Transition speed of AuNP-doped TiNS-Gel from TiNS-Gel$_{\mathrm{Repuls}}$ to TiNS-Gel$_{\mathrm{Attract}}$ by photoirradiation.

a, A schematic illustration for photoinduced transition of AuNP-doped TiNS-Gel from TiNS-Gel$_{\mathrm{Repuls}}$ to TiNS-Gel$_{\mathrm{Attract}}$. **b**, **c**, Time-course plots of the normalized gray value of the POM image of AuNP-doped TiNS-Gel ([TiNS] = 14 wt%) upon photoirradiation. The hydrogel filled in a 0.2-mm-thick glass container was irradiated with 445-nm laser lights (irradiated region = 2 × 4 mm) with various light power densities (0.56–5.6 W cm^{-2}) at a fixed AuNP concentration of 0.05 wt% (**b**) and with various AuNP concentrations (0.005–0.05 wt%) at a fixed light power density of 5.6 W cm^{-2} (**c**).

Supplementary References

1. Tanaka, T., Ebina, Y., Takada, K., Kurashima, K. & Sasaki, T. Oversized titania nanosheet crystallites derived from flux-grown layered titanate single crystals. *Chem. Mater.* **15**, 3564–3568 (2003).
