# Supplementary material for: A mechanically adaptive hydrogel with a reconfigurable network consisting entirely of inorganic nanosheets and water
Source: Nat Commun. 2020 Nov 27;11:6026. doi: 10.1038/s41467-020-19905-4 (PMC7699623; doi:10.1038/s41467-020-19905-4)
Supplement: Supplementary file 3 — Description of Additional Supplementary Files [file 41467_2020_19905_MOESM3_ESM.pdf]

Description of Additional Supplementary Files

File Name: Supplementary Movie 1

Description: **Photoinduced spatiotemporal gel-to-gel transition of TiNS-Gel.**

Changes in polarized optical microscopic images under crossed Nicols of TiNS-Gel doped with AuNP as a photothermal converter ([TiNS] = 14 wt%; [AuNP] = 0.05 wt%), which was filled in a 0.2-mm-thick glass container. When TiNS-Gel$_{Repuls}$ was irradiation with the 445-nm laser light (power density = 5.6 W cm^{-2}; irradiated region = 2 × 4 mm) for 40 s, the irradiated region was selectively transformed into TiNS-Gel$_{Attract}$ with no birefringence. Upon ceasing photoirradiation, the irradiated region was air-cooled and returned to TiNS-Gel$_{Repuls}$ with a large birefringence within 4 s. This cycle could be repeated many times without deterioration.
